# Acute pancreatitis presented with diffuse ST-segment elevation: A case report and literature review

**DOI:** 10.1097/MD.0000000000037245

**Published:** 2024-02-16

**Authors:** Yi-Lin Hsieh, Shu-Hao Wu, Chia-Yuan Liu, Wei-Chen Lin, Ming-Jen Chen, Chen-Wang Chang

**Affiliations:** a MacKay Medical College, New Taipei City, Taiwan; b Department of Internal Medicine, MacKay Memorial Hospital, Taipei, Taiwan; c Division of Cardiology, Department of Internal Medicine, MacKay Memorial Hospital, Taipei, Taiwan; d Division of Gastroenterology, Department of Internal Medicine, MacKay Memorial Hospital, Taipei, Taiwan; e MacKay Junior College of Medicine, Nursing and Management, Taipei, Taiwan.

**Keywords:** acute pancreatitis, ECG

## Abstract

**Introduction:**

Although electrocardiographic changes have been previously reported in patients with acute pancreatitis, diffuse ST-segment elevation without occluded coronary arteries is rarely documented.

**Patient concerns:**

A 45-year-old man presented to our emergency department due to persistent epigastric pain for 2 hours. However, ECG in the emergency department revealed regular sinus rhythm at 67 beats per minute, peaked T waves in lead V3-5, and upsloping ST-segment elevation in leads II, III, aVF, and V2-6.

**Diagnosis:**

He was diagnosed with acute pancreatitis and presented with diffuse ST-segment elevation.

**Interventions:**

Laboratory workup and computed tomography supported the diagnosis of acute gallstone pancreatitis and endoscopic retrograde cholangiopancreatography was performed. Coronary angiography showed patent coronary arteries finally.

**Outcomes:**

Endoscopic retrograde cholangiopancreatography and endoscopic papillo-sphincterotomy were performed, and the stone in the common bile duct was removed smoothly without immediate complication. Due to his relatively stable condition, he was discharged on day 7 of admission.

**Conclusion:**

We presented an uncommon case of acute pancreatitis demonstrating similar features of AMI. This reminds cardiologists and emergency physicians to make the judgment with more caution to avoid jumping to conclusions and providing inappropriate treatment.

## 1. Introduction

Acute pancreatitis is an acute inflammatory process of the pancreas that can demonstrate variable degrees of involvement in one or more organ systems outside of the pancreas. The disease has been reported to be accompanied by several different abnormal ECG changes,^[1]^ most frequently associated with T-wave inversion and ST-segment depression.^[2]^ Most changes are transient and subside spontaneously after the condition is sufficiently stabilized, albeit the underlying pathogenesis remains unclear. There are a few documented cases of acute pancreatitis that presented with abnormal ECG changes and elevated cardiac enzymes that mimicked life-threatening acute myocardial infarction (AMI) but showed patent coronary arteries on angiography later in the clinical course, excluding the possibility of myocardial infarction with coronary athero-thrombosis.^[3–8]^ Although acute pancreatitis is rarely complicated by true ST-elevation myocardial infarction (STEMI), ST-segment elevation is an important cause for concern and should be emergently evaluated as it demands a different approach to treatment than that of acute pancreatitis. We present a case of epigastric pain with anterior and inferior wall ST-segment elevation on ECG who was diagnosed with patent coronary arteries and acute pancreatitis.

## 2. Case report

A 45-year-old man presented to our emergency department (ED) due to persistent epigastric pain for 2 hours, accompanied by abdominal bloating sensation, nausea, and vomiting. The pain was dull and radiated to the back. The patient denied chest tightness, dyspnea, and diaphoresis. Besides, he came to our ED and left against medical advises for similar complaints 2 days prior to this visit. The patient was an active smoker (0.5 pack-per-day for 15 years) but denied alcohol intake. He had a medical history of hypertension without treatment and hepatitis B virus infection. His vital signs were stable with regular heart rate of 68 beats per minute, afebrile body temperature of 36 ºC, blood pressure of 141/83 mm Hg, and saturated 100% on room air. On physical examination, jaundice and severe abdominal tenderness over the epigastric region were observed without obvious rebound tenderness or abdominal muscle guarding.

**Figure 1.**
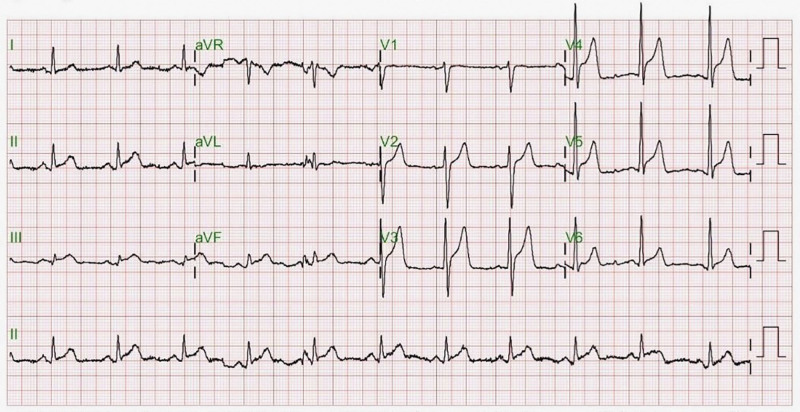
First electrocardiogram recorded at triage, revealing anterior and inferior wall ST-elevation.

ECG in the ED revealed regular sinus rhythm at 67 beats per minute, peaked T waves in lead V3-5, and upsloping ST-segment elevation in leads II, III, aVF, and V2-6 (Fig. 1). With the concern for STEMI, the cardiologist was consulted for potential emergency cardiac catheterization. Bedside echocardiography showed good left ventricle systolic function without regional wall motion abnormalities. The creatine kinase (CK, 438 IU/L) and CK-MB (11.0 ng/mL) levels were elevated but the troponin-I level remained within normal limits (<0.01 ng/mL) at the time of arrival at our ED; all biomarkers returned to baseline 4 hours later.

**Figure 2.**
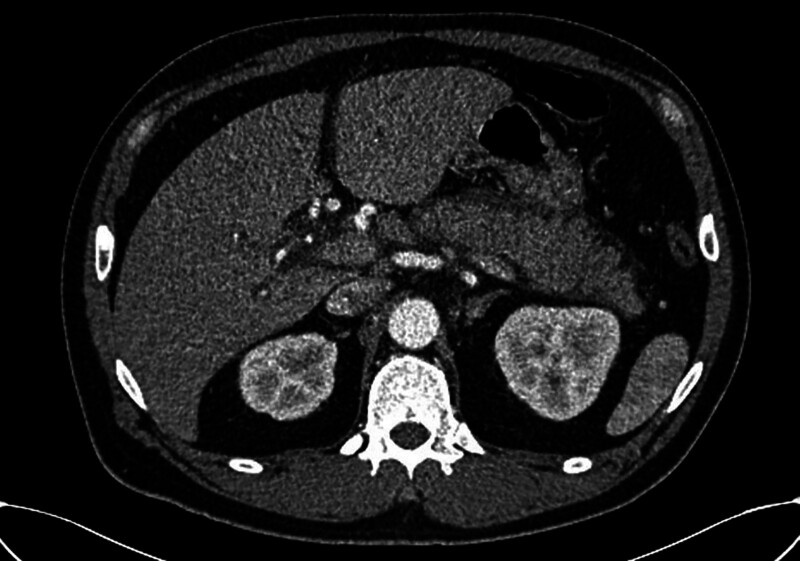
Abdominal CT showing edematous change in the left anterior segment of pancreas, suggestive of acute pancreatitis.

Abdominal computed tomography scan showed choledocholithiasis with obstructive cholangitis in the dilated common bile duct and edematous change of pancreas, suggestive of acute pancreatitis (Fig. 2). Leukocytosis with WBC 13,700 cells/μL, and biochemical laboratory showed acute hepatitis and cholangitis (AST 359 IU/L, alanine transaminase 800 IU/L), elevated total (13.4 mg/dL) and direct bilirubin (8.8 mg/dL). Elevated amylase (4368 U/L) and lipase (>2000 U/L) were also found, and the tentative diagnosis of acute biliary pancreatitis was made. During admission, the follow-up blood tests demonstrated an elevation in troponin-I (1.791 ng/mL) on day 2 but ECG showed normal sinus rhythm without other pertinent findings. He was free of symptoms and denied chest tightness and shortness of breath. The following coronary angiography revealed patent coronary arteries and coronary artery ectasia with slow flow.

Endoscopic retrograde cholangiopancreatography (ERCP) and endoscopic papillo-sphincterotomy were performed 1 day later, and the stone in the common bile duct was removed by balloon extraction smoothly without immediate complication. The patient could tolerate a diet well 1 day after the procedure. Follow-up blood tests showed that liver enzymes, amylase, lipase, and cardiac troponin levels all returned to normal range after ERCP. Due to his relatively stable condition, he was discharged on day 7 of admission.

## 3. Discussion

According to the guidelines^[9]^ and Revised Atlanta Classification,^[10]^ the diagnosis of acute pancreatitis can be reliably established by the presence of 2 of the 3 following criteria: characteristic abdominal pain consistent with the disease, serum amylase or lipase more than 3 times the upper limit of normal, and characteristic findings on abdominal imaging. In our case, he was diagnosed with acute pancreatitis, but the concerning presentation of abnormal ECG and increased cardiac biomarkers that mimic life-threatening AMI poses a major diagnostic and treatment challenge.

A nationwide cohort study indicated that the rate of AMI in patients with acute pancreatitis is 5.44 per 1000 person-years^[11]^; however, pseudo-STEMI has been described as a complication of acute pancreatitis for 50 years.^[1,4]^ Increased cardiac biomarkers such as cardiac troponins typically found in myocardial injuries should provide additional evidence and elicit the diagnosis, but there are also documented cases of acute pancreatitis demonstrating an elevation of troponin level,^[3,4]^ complicating the subsequent treatment strategies.

Currently, the underlying mechanism linking myocardial injuries to acute pancreatitis remains unclear. Nonetheless, there have been multiple proposed hypotheses that tried to explain this phenomenon. (1) Transient development of thrombus to produce myocardial ischemia secondary to the hemostatic and fibrinolytic abnormalities of acute pancreatitis.^[12]^ (2) Stress-induced by critical illness of acute pancreatitis results in a transient dysfunction of the left ventricular apex, also known as Takotsubo cardiomyopathy.^[8,13]^ (3) Proteolytic enzymes, especially trypsin, are activated and released into the bloodstream, inducing digestion of myocardial cellular membranes and ultimately myocardial necrosis.^[3,5,14]^ (4) Increased autonomic vagal tone, or “cardiobiliary reflex,” leading to transient coronary spasm and AMI-like features.^[4,15]^ Yu et al reported 32 cases of acute pancreatitis that demonstrates similar ECG features mimicking that of AMI among which ECG patterns of inferior wall infarction is the most common,^[4]^ favoring trans-diaphragmatic passage of epicardial inflammation as the underlying pathogenesis due to the relative retroperitoneal location of the organ in relation to the heart. Nevertheless, neither isolated inferior MI-like ECG features nor evident occluded coronary arteries were presented in our case. This atypical presentation emphasizes the importance of acute pancreatitis and diffuse ST-segment elevation, especially in precordial leads.

According to the Fourth Universal Definition of Myocardial Infarction,^[16]^ the term “myocardial injury” is defined by a definite increase of cardiac troponin upwards of the 99th percentile upper normal limits. In the present case, the patient did not meet the corresponding criteria, denoting that the presence of the ECG changes at the time of admission did not necessarily correspond to myocardial injury; without pertinent findings detected with bedside echocardiogram, it may be secondary to a transient pancreatitis-induced shift in equilibrium of oxygen supply and demand. The rise of cardiac troponin 2 days after this admission satisfies the criteria for myocardial infarction, indicating that deterioration of present pancreatitis without definite treatment contributed to the tilted balance of myocardial perfusion eventually accumulated into biochemically defined myocardial injury. The restoration of cardiac troponin levels after removal of gallstone via ERCP provided additional evidence that pancreatitis was the culprit lesion in this very scenario.

In order to meet the critical door-to-balloon time in an emergent scenario of STEMI, an examination of cardiac troponin level is unlikely to be obtained at the time of triage. Additional noninvasive testing can help to guide clinical decision-making: observation of dynamic ECG changes and point-of-care echocardiography are both endorsed by current acute coronary syndrome guidelines.^[17–19]^ In the present case, the absence of both trans-thoracic echocardiographic and serial ECG abnormalities suggests that the presence of an episode of acute cardiac insult was less likely. Thus, emergent catheterization was postponed.

In summary, we presented an uncommon case of acute pancreatitis demonstrating similar features of AMI. Although previous hypotheses suggested acute pancreatitis may cause coronary artery disease by influencing hemostasis and ST-segment elevation were observed most in inferior leads due to trans-diaphragmatic passage of epicardial inflammation, our case presented with diffuse ST-segment elevation and elevated cardiac enzyme without occluded coronary arteries. This remind cardiologists and emergency physicians to make the judgment with more caution to avoid jumping to conclusion and providing inappropriate treatment.

## Author contributions

**Conceptualization:** Ming-Jen Chen.

**Data curation:** Ming-Jen Chen.

**Formal analysis:** Wei-Chen Lin.

**Investigation:** Shu-Hao Wu.

**Validation:** Shu-Hao Wu, Wei-Chen Lin, Chia-Yuan Liu.

**Writing – original draft:** Yi-Lin Hsieh.

**Writing – review & editing:** Chen-Wang Chang.
